# Difficulties in Generating Specific Antibodies for Immunohistochemical Detection of Nitrosylated Tubulins

**DOI:** 10.1371/journal.pone.0068168

**Published:** 2013-06-28

**Authors:** Anton Kamnev, Matthias Muhar, Martina Preinreich, Hermann Ammer, Friedrich Propst

**Affiliations:** 1 Max F. Perutz Laboratories, University of Vienna, Department of Biochemistry and Cell Biology, Vienna, Austria; 2 Institute of Pharmacology, Toxicology and Pharmacy, Ludwig-Maximilians University Munich, Munich, Germany; Albany Medical College, United States of America

## Abstract

Protein S-nitrosylation, the covalent attachment of a nitroso moiety to thiol groups of specific cysteine residues, is one of the major pathways of nitric oxide signaling. Hundreds of proteins are subject to this transient post-translational modification and for some the functional consequences have been identified. Biochemical assays for the analysis of protein S-nitrosylation have been established and can be used to study if and under what conditions a given protein is S-nitrosylated. In contrast, the equally desirable subcellular localization of specific S-nitrosylated protein isoforms has not been achieved to date. In the current study we attempted to specifically localize S-nitrosylated α- and β-tubulin isoforms in primary neurons after fixation. The approach was based on *in situ* replacement of the labile cysteine nitroso modification with a stable tag and the subsequent use of antibodies which recognize the tag in the context of the tubulin polypeptide sequence flanking the cysteine residue of interest. We established a procedure for tagging S-nitrosylated proteins in cultured primary neurons and obtained polyclonal anti-tag antibodies capable of specifically detecting tagged proteins on immunoblots and in fixed cells. However, the antibodies were not specific for tubulin isoforms. We suggest that different tagging strategies or alternative methods such as fluorescence resonance energy transfer techniques might be more successful.

## Introduction

Nitric oxide (NO) is a well-established neuromodulator and neurotransmitter in the central and peripheral nervous systems [1] and has been shown to be involved in the modulation of synaptic efficacy, pain perception and neuronal damage/protection [2]. NO acts mainly through activation of cGMP signaling [3] or through S-nitrosylation of proteins at specific cysteine residues [4], [5]. Over the last decade hundreds of proteins have been shown to be S-nitrosylated [6], [7]. Functional consequences of S-nitrosylation have been demonstrated for a small number of proteins, including caspases [8], parkin [9], glyceraldehyde 3-phosphate dehydrogenase (GAPDH) [10], tubulin [11], microtubule-associated protein MAP1B [12], histone deacetylase-2 (HDAC2) [13], PSD-95 [14] and AMPA receptors [15]. However, for most of the identified targets, the exact role and relevance of S-nitrosylation remain elusive.

A major obstacle in the analysis of protein S-nitrosylation is the low stability of this posttranslational modification in reducing environments and upon exposure to light [16]. This problem was partially overcome by the development of the biotin-switch procedure [5]. In this biochemical assay the unstable nitroso moiety of S-nitrosylated cysteine residues is replaced by a stable biotin tag. This replacement is not target specific. In theory, all S-nitrosylated cysteine residues in a biological protein lysate will be labeled by the method.

The biotin switch protocol represented a breakthrough, facilitating biochemical analysis of protein S-nitrosylation. On the other hand, it would be equally desirable to determine the subcellular localization of S-nitrosylated protein species. To this end the biotin switch protocol was adapted to biotin-label S-nitrosylated cysteine residues *in situ* [17], [18]. This permitted the detection of S-nitrosylated proteins in fixed cells using fluorescently labeled streptavidin. A major drawback of this method is that it is not specific for a given protein and indiscriminately detects all cellular proteins with S-nitrosylated cysteine residues.

In the current study we aimed to develop a procedure for specifically detecting individual S-nitrosylated protein species in fixed primary neurons. We chose α- and β-tubulin as target proteins. Both proteins are S-nitrosylated in the murine brain [5]–[7]. Moreover, tubulin and microtubules are subject to diverse post-translational modifications, the subcellular localization of which has yielded important insights concerning their function [19]. Our approach was based on raising antibodies against synthetic biotin-tagged α- or β-tubulin peptide epitopes in the hope that these antibodies will specifically recognize biotin-tagged cysteine residues only in the context of the α- or β-tubulin polypeptide sequence. We succeeded in biotin-tagging S-nitrosylated proteins in fixed primary neurons and obtained antisera recognizing biotin-tagged cysteine residues. However, the antisera were not specific for tubulin isoforms.

## Materials and Methods

### Ethics Statement

Tissues from mice were obtained in compliance with the Austrian law regulating the use of animals in biomedical research, Tierversuchsgesetz, BGBl. Nr. 501/1989 and BGBl. I Nr. 162/2005.

### Commercial Antibodies and Detection Reagents

Primary antibodies: goat anti-biotin antibodies conjugated to alkaline phosphatase (Abcam, Cambridge, UK); mouse anti-pan-α-tubulin monoclonal antibodies (clone B-5-1-2; Sigma, St. Louis, MI), rabbit anti-pan-β-tubulin polyclonal antibodies (Abcam).

Secondary antibodies: Alexa Fluor 488-labelled goat anti-rabbit antibodies (Invitrogen, Carlsbad, CA); horse radish peroxidase-conjugated goat anti-rabbit (Vector Laboratories, Burlingame, CA) or anti-mouse antibodies (Jackson ImmunoResearch Laboratories, West Grove, PA) or alkaline phosphatase-conjugated goat anti-rabbit or anti-mouse antibodies (Jackson).

Rhodamine-red- (Jackson) and horse radish peroxidase-labeled (Abcam) streptavidin.

### Immunogen Synthesis and Production of Antisera

The positions of tubulin cysteine residues S-nitrosylated *in vivo* in the α/β-tubulin heterodimer were determined using RasMol software. The 3D structure of the α/β-tubulin heterodimer was taken from the protein data bank (PDB ID: 1TUB) [20]. The selection of peptide sequences flanking the cysteines of interest was based on the sequences of α-tubulin (a1Tub; NP_071634.1) and β-tubulin (b5Tub; NP_035785.1), respectively. The peptides to be synthesized were VAEITNACFEPANQM (immunogen-α) and KNMMAACDPRHGR (immunogen-β).

Peptides were synthesized by INTAVIS AG (Reutlingen, Germany) using the Fmoc solid-phase technology, purified by HPLC (>90%) and analyzed by MALDI-TOF mass spectrometry for integrity. For immunization, peptides were coupled through their internal free SH-group to primary amino-groups of keyhole limpet hemocyanin carrier protein (KLH; Calbiochem, Darmstadt, Germany) by a two-step method using the heterobifunctional cross-linker LC-SPDP (Thermo Fisher Scientific Inc., Waltham, MA) essentially as described [21]. In reaction A, iodoacetamide-treated KLH (10 mg) was modified with LC-SPDP (12.8 mg) for 30 min at room temperature in a total volume of 2.5 ml of 0.1 M sodium phosphate buffer, pH 7.5, containing 0.15 M NaCl and 1 mM EDTA (PBS-EDTA). The resulting pyridyldithio-activated carrier intermediate was then purified by gel filtration chromatography over Sephadex G-25 (GE Healthcare, Pittsburg, PA), split into two aliquots of 1.75 ml containing 5 mg of activated carrier in PBS-EDTA. To each vial 5 mg of Immunogen-α or Immunogen-β solubilized in PBS-EDTA was added and reacted overnight at 4°C (reaction B). Non-reacted N-hydroxysuccinimide ester was quenched by the addition of 1 mg of cysteine and incubation for 30 min at room temperature and coupling efficiency was estimated by determination of pyridine 2-thione released as described below and was almost complete. The final protein-peptide conjugates were dialyzed into 0.9% NaCl solution to remove salts and non-conjugated peptides and stored in aliquots at −20°C. A summary of the coupling procedure is given in Figure S1A. Two rabbits were immunized with each immunogen. Sera were collected according to standard procedures (Gramsch Laboratories, Schwabhausen, Germany).

### Blocking of the Antisera with Tagged Peptides

40 µl of a 2 mg/ml solution of the corresponding peptide in phosphate buffered saline (PBS) containing 1 mM EDTA (Gerbu, Wieblingen, Germany) was mixed with the appropriate amount (5–6 µl) of 5 mM biotin-HPDP (Thermo Fisher Scientific Inc.) solution in DMSO (Sigma) in order to achieve a final molar ratio of 1∶1 of peptide to biotin-HPDP (final peptide concentration 1 mM). The mixture was left at room temperature in the dark for 30 min. A separate identical mixture was used to monitor the course of the reaction at a wavelength of 343 nm (absorbance of pyridine-2-thione, a byproduct of the tagging reaction). This allowed calculating that at least 30–50% of input peptide was successfully tagged. After incubation, 20 µl of the mixture was added to 1.5 ml of 2% bovine serum albumin (BSA; PAA Laboratories GmbH, Cölbe, Germany) in PBST (0.25% Tween-20 (Gerbu) in PBS). The excess of BSA neutralizes unreacted biotin-HPDP in the mixture. Finally 3 µl of corresponding antiserum was added and incubated over night at 4°C with constant mixing. With a measured concentration 2.3 µM of IgG antibodies reactive with tagged tubulin peptide (4.6 µM of epitope binding sites) in the serum the final molar excess of tagged peptide over antibody epitope binding sites is at least 500-fold. In addition, we confirmed proper formation of inhibitor-TPα by mass spectrometry (not shown).

### Cell Culture

Adult dorsal root ganglion (DRG) neurons were prepared as previously described [12]. Neurons were grown on glass coverslips for 48 h before treatment and/or fixation. Cortical and hippocampal neurons were obtained from newborn mice. Following decapitation the neocortex was separated from the rest of the brain and meninges were removed in Hank’s balanced salt solution (HBSS, Invitrogen) with 1.3 mM Ca^2+^ and 1 mM Mg^2+^, 20 mM Hepes (Gerbu), 50 U/ml penicillin, 50 µg/ml streptomycin and 1× glutamax (Invitrogen) on ice. The isolated neocortex was minced to pieces of approximately 2 mm in diameter and treated with trypsin 0.25%, 0.7 mM EDTA in HBSS (without calcium and magnesium), 5 ml per brain, at 37°C for 25 min with occasional stirring. The medium was removed and the pieces were incubated for 5 min in Neurobasal medium A (Invitrogen) supplemented with 20% of heat inactivated horse serum (Invitrogen; 3 ml per brain). Next the tissue was disrupted by gentle trituration with a fire polished Pasteur pipette. Cells were collected by centrifugation at 1000×g for 3 min, washed once with growth medium (Neurobasal medium A supplemented with 1× glutamax (Invitrogen), 1× B27 supplement (Invitrogen), 50 U/ml penicillin (Invitrogen) and 50 µg/ml streptomycin (Invitrogen), re-suspended in growth medium (4 ml per one brain) and filtered through 70-µm and 40-µm nylon cell strainers (BD Biosciences, San Jose, CA) to remove tissue debris. After filtering, the concentration of the cell suspension was determined by counting cells with a hemocytometer (1 brain on average yielded 3×10^6^ neurons). Finally, neurons were plated at a density of 2×10^4^ cell/cm^2^ on poly-L-lysine (100 µg/ml; Sigma) and laminin I (100 µg/ml; Sigma) coated glass coverslips and cultivated for up to 4 days at 37°C in a humidified atmosphere containing 5% CO_2_.

For *in situ* analysis of protein S-nitrosylation N2a and COS7 cells were grown on glass coverslips coated with poly-L-lysine (10 µg/ml) and laminin I (100 µg/ml) at 37°C in a humidified atmosphere containing 5% CO_2_ in Dulbecco’s modified Eagle’s medium containing 25 mM D-Glucose (Invitrogen), 50 U/ml penicillin, 50 µg/ml streptomycin, 2.5 µg/ml fungizone (Invitrogen), 2 mM L-glutamine (Invitrogen) and 10% fetal calf serum (Sigma). For the preparation of cell lysates N2a cells were seeded onto plastic culture dishes at a density of 3×10^4^ cells/cm^2^ and grown under the same conditions.

### Preparation of Brain and N2a Cell Lysates for Biotin Switch Assay *in vitro*

Fresh adult mouse brain samples were homogenized in HEN/10 buffer (2.5 mM Hepes pH 7.7, 0.01 mM EDTA and 1 nM neocuproine (Sigma)) and cleared by centrifugation at 3000×g for 15 min. The resulting supernatant routinely contained proteins at a concentration of 1 to 2 mg/ml. Brain lysates were used fresh. For N2a cell lysates, the growth medium was replaced by differentiation medium (as above, but without fetal calf serum and instead containing 3 mM dibutyryl-cyclic AMP (Sigma)) one day after plating and the cells were incubated for an additional 2 days. Cells were rinsed once with HEN/10 buffer and then kept on ice for 3 min in a minimal amount of HEN/10 supplemented with protease inhibitors (Roche, Basel, Switzerland). Cells were scraped off the plate and sonicated on ice. The lysate was cleared by centrifugation at 7000×g for 10 min at 4°C. The resulting supernatant routinely contained proteins at a concentration of 1 to 2 mg/ml. The lysate was used either fresh or was frozen immediately in liquid nitrogen and stored (for not more than 1 week) at −70°C for later use.

### Biotin Switch Protocol

Treatment of brain and N2a cell lysates with GSNO or SNAP to S-nitrosylate cysteine residues in target proteins was carried out as described [5]–[7], [22]. The analysis of protein S-nitrosylation in brain lysates was carried out using a biotin switch protocol in combination with resin-assisted capture (SNO-RAC) developed by Forrester et al. [23]. The *in vitro* biotin switch assay using N2a cell lysates was carried out as described [5], except that the resulting biotinylated proteins were directly analyzed by immunoblotting. The *in situ* analysis of protein S-nitrosylation was based on the protocol of Yang and Loscalzo [18]. Briefly, cells grown on glass coverslips were treated for 20 min with S-nitrosoglutathione (GSNO) prepared as described [24] or S-nitroso-N-acetyl-D,L-penicillamine (SNAP; Invitrogen) dissolved in DMSO. Control samples were treated either with glutathione (GSH; Sigma) or were left untreated. Following treatment, cells were fixed in −20°C methanol (VWR, Vienna, Austria) and kept at -20°C for 10 min. After fixation, methanol was discarded and blocking solution consisting of 200 mM S-methyl methanethiosulfonate (MMTS; Sigma) in HEN/methanol (100 mM Hepes pH 7.7, 1 mM EDTA, 0.2 mM neocuproine in 80% methanol) was added. Free thiol residues were blocked by incubation for 30 min at 55°C with gentle shaking. After the incubation coverslips were washed 3 times for 5 min in fresh HEN/methanol to remove excess MMTS. S-nitrosothiols were reduced with 0.2 mM Na-ascorbate and labeled with 0.2 mM biotin-HPDP (1 h at room temperature). Coverslips were washed again 3 times for 5 min with HEN/methanol, rinsed once with PBST and incubated with 2% BSA in PBST for 30 min. Subsequently, cells were stained with streptavidin-rhodamine red or primary antibodies in PBST for 1 h at room temperature, washed 3 times for 5 min with PBST and incubated for 1 h with appropriate secondary antibodies in PBST. Cells were washed twice for 5 min in PBST, once for 5 min in PBS, rinsed in H_2_O, desiccated by passing them through 70% and 96% ethanol and mounted.

### Immunoblot Detection of Proteins

Biotinylated proteins were fractionated by non-reducing SDS–PAGE and transferred to nitrocellulose membranes (Whatman, Maidstone, UK). Membranes were blocked in PBST containing 2% BSA and 5% skim milk (Gerbu) for 1 h at room temperature (or overnight at 4°C). Incubation with appropriate primary antibodies was done in PBST containing 2% BSA for 1.5 h at room temperature (or overnight at 4°C). The membranes were washed 3 times for 5 min with PBST. After washing, the membranes were incubated with the appropriate alkaline phosphatase- or horse radish peroxidase-conjugated secondary antibodies for 1 h at room temperature and developed using standard methods.

### Fluorescent Microscopy and Image Quantification

Microscopy was performed either on a laser scanning confocal microscope LSM510 (Zeiss, Oberkochen, Germany) or an API personal Deltavision system (Applied Precision, Issaquah, WA). For quantification of fluorescence we used ImageJ (rsbweb.nih.gov). Quantification was performed on cell bodies (N2a cells, DRG, cortical and hippocampal neurons) since processes very often retract during methanol fixation and show a very low level of signal with intensities similar to the background. In COS7 cells the fluorescent signal was measured over the entire cell. For each group cells were imaged from at least two independent experiments with two independent treatments each. From each single treatment (1 coverslip with cells) 10 to 15 images were taken. 10 cells were randomly selected for analysis and the average intensity of the fluorescence signal was determined for each cell.

### Statistical Analysis

First, for each control group a median value of fluorescence intensity was calculated. Then, for each cell in both experimental and control groups the average intensity values were normalized to the median value for the control group. Finally, the statistical significance of differences in median values of intensity in control and experimental groups was determined using the independent samples Kruskal-Wallis test with a significance level of 0.05. The analysis was performed using the SPSS software (IBM, New York, NY).

## Results

### Strategy for Immunological Detection of Tubulin S-nitrosylation in Fixed Cells

**Figure 1:**
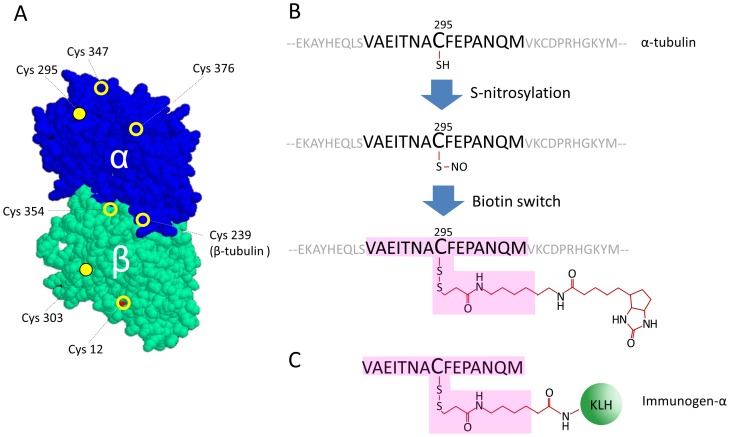
Concept for specific detection of S-nitrosylated tubulin by a combination of the biotin switch protocol and immunodetection. (A) 3D structure of the α/β-tubulin heterodimer [20]. Positions of cysteine residues found to be S-nitrosylated in murine brain are marked. Cysteine residues are localized on the surface of the dimer (filled circles) as well as inside the polypeptides (open circles). Cys 295 and Cys 303 of α- and β-tubulin, respectively, were chosen for further analysis. (B) Tagging of S-nitrosylated tubulin. Endogenously or exogenously S-nitrosylated cysteine residues (shown here for Cys 295 of α-tubulin) are stably tagged with biotin-HPDP using the biotin switch protocol. (C) Structure of immunogen-α. Pink shading outlines the epitope of interest.

Mass spectrometric analysis of protein S-nitrosylation in the murine brain revealed both α- and β-tubulin to be S-nitrosylated on 3 and 4 cysteine residues, respectively [6],[7] (Fig. 1A). Posttranslational modifications of tubulin often display a specific subcellular localization in neurons. Therefore, we wanted to determine whether S-nitrosylation of tubulin is also restricted to subcellular regions.

We decided to use an approach based on the immunological detection of specific protein epitopes carrying the modification. This approach has been used successfully, for example, for the specific detection of proteins phosphorylated at a given epitope. However, in contrast to phosphorylation, the modification of cysteine residues by S-nitrosylation is highly unstable, with a half-life in the range of seconds to minutes [25]. This necessitated the development of an indirect approach which was based on the biotin switch method [5]. This technique allows replacement of the labile S-nitroso modification of a given cysteine with a stable tag, for example the biotin-HPDP tag (Fig. 1B). The biotin switch protocol was originally designed for protein lysates of tissues or cultured cells, but recently has been adapted to tag proteins in fixed cells as well [17],[18]. We reasoned that proteins containing S-nitrosylated cysteine residues in live cells could be fixed, tagged *in situ* by biotin-HPDP and detected and localized by antibodies specific for the tagged cysteine residue in the context of the polypeptide chain. In addition, we surmised that synthetic peptides with a sequence corresponding to a region flanking an S-nitrosylated cysteine of interest and containing a stable tag on the cysteine could be used as immunogens to produce protein context-specific anti-tag antibodies.

We chose LC-SPDP (Fig. S1A) as tag to generate the immunogens for several reasons: (i) LC-SPDP and biotin-HPDP share the molecular structure at the thiol-modifying end. Thus, antibodies raised against cysteine residues carrying the LC-SPDP tag can be expected to recognize proteins modified by the biotin-HPDP tag used in the biotin switch protocol (Fig. 1B and C, pink shading); (ii) LC-SPDP does not contain the highly immunogenic biotin moiety and therefore is less likely to induce tag-only antibodies; (iii) in addition to the pyridine 2-thionyl group reactive with thiol groups of cysteine residues, LC-SPDP contains an amine-reactive *N*-hydroxysuccinimide ester group that can be used for directly coupling a peptide epitope containing a cysteine residue to lysine residues of a carrier protein (Fig. S1A). Antisera raised against such immunogens should be able to specifically bind to biotin-HPDP modified cysteine residues in a specific context within a polypeptide, and thus, could be used for detection of specific S-nitrosylated proteins *in situ*.

Analysis of the available structure of the α/β-tubulin heterodimer in the context of microtubules showed that only two cysteine residues are localized at the surface of the microtubule: Cys 295 of α-tubulin and Cys 303 of β-tubulin (Fig. S1B). Alignment of different isoforms of mouse tubulins showed that the amino acid sequences flanking Cys 295 and Cys 303 are well conserved. We chose to focus on these two cysteines, since their surface localization was expected to facilitate detection with antibodies.

### Characterization of Antisera Raised Against Tagged Tubulin Peptides by Immunoblotting

**Figure 2:**
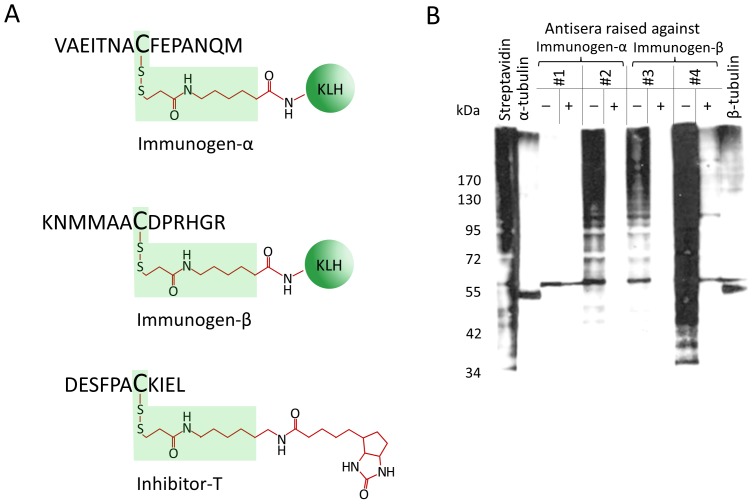
Characterization of antisera raised against tagged tubulin peptides. (A) Schematic representation of immunogens and inhibitor-T. Antibodies directed against an epitope contained within the area shaded in green would detect either just the tag or tagged cysteines in a context-unspecific manner. The reactivity of these antibodies can be blocked by inhibitor-T, an unrelated peptide carrying the biotin tag on its internal cysteine. (B) Lysates of differentiated N2a cells were treated with 100 µM GSNO for 20 min and then subjected to the biotin switch protocol. 17.4 µg of biotinylated lysate was loaded in each lane, fractionated by non-reducing SDS-PAGE and transferred to a nitrocellulose membrane. Following blocking the membrane was cut into stripes and each stripe was then probed with the indicated antiserum diluted at 1∶500 pre-incubated in the presence (*+*) or absence (−) of inhibitor-T. Successful application of the biotin switch protocol was confirmed with streptavidin-HRP (*streptavidin*) and the positions of tubulin bands were located with both anti-α- (*α-tubulin)* and anti-β-tubulin (*β-tubulin*) antibodies as indicated. Loss in staining after pre-incubation with inhibitor-T of serum #2, #3 and #4 shows that these antisera contain mostly context-unspecific antibodies directed against the tag or tagged cysteine. However, antisera #1 and #4 also bind to a 60-kDa band, the detection of which is insensitive to pre-incubation of the antisera with the non-specific inhibitor-T. This band co-migrated with the upper band detected with pan anti-β-tubulin antibodies in lysates that were subjected to S-nitrosylation and the biotin switch protocol.

The immunogens used for immunization, the synthetic, tagged tubulin peptides immunogen-α and immunogen-β (Fig. 2A) can potentially give rise to three types of antibodies. The goal of our study was to obtain context-specific antibodies which recognize the tagged cysteine residue only in the context of the amino acid sequence of the synthetic tubulin peptide. However, the immunogens can also induce two types of unwanted antibodies which would yield false positive results: (i) antibodies recognizing the synthetic peptide both in the tagged and non-tagged form (anti-peptide antibodies) and (ii) antibodies recognizing just the tag or tagged cysteine, regardless of the amino acids flanking the cysteine residue (context-unspecific antibodies; Fig. 2A, green shading).

Positive purification of the desired context-specific antibodies is not possible, because anti-peptide antibodies as well as context-unspecific antibodies are bound to co-purify on columns carrying the immobilized immunogen. An alternative strategy to increase specificity of the antisera is to block the reactivity of potential unwanted anti-peptide and context-unspecific antibodies. We employed this strategy and first tested the reactivity of the antisera by immunoblot assays. For this purpose we generated two types of protein lysates of N2a cells. One type consisted of whole cell lysates obtained under reducing conditions. These lysates will only contain reduced and hence non-nitrosylated proteins including tubulins. Immunoblot testing of the antisera using reduced whole cell lysates revealed that there were only trace amounts of anti-peptide antibodies present in the sera (not shown) obviating the need to block them. To test the reactivity of the sera against nitrosylated proteins that were biotin-tagged in the course of the biotin switch protocol we used a second type of protein lysates. These lysates were treated with GSNO *in vitro* to nitrosylate target cysteines in cellular proteins including tubulin prior to application of the biotin switch protocol. *In vitro* GSNO treatment of protein lysates has been used successfully to generate α- and β-tubulin S-nitrosylated at the cysteine residues of interest [5]–[7],[22]. In addition, we verified that *in vitro* nitrosylation of protein lysates with another NO donor, SNAP, also lead to nitrosylation of tubulin (Fig. S2). Using GSNO treated lysates for immunoblot analyses we found that three out of the four antisera (antisera #2, #3 and #4) reacted with tag only or with tagged cysteines regardless of context. This was evident from their reactivity on immunoblots with a wide variety of S-nitrosylated proteins of different sizes, provided that gel electrophoresis was carried out under non-reducing conditions (Fig. 2A), but not under reducing conditions (not shown). To neutralize the reactivity of these context-unspecific antibodies we incubated the antisera with an excess of inhibitor-T consisting of a short peptide unrelated to tubulin with a single internal cysteine that is modified with biotin-HPDP. This tagged peptide should neutralize antibodies which recognize tag only or tagged cysteines regardless of the flanking amino acid sequence (green shading, Fig. 2A). Indeed, pre-incubation of antisera #2 and #3 blocked all reactivity on immunoblots, indicating that these antisera contained only context-unspecific anti-tag antibodies (Fig. 2B).

Anti-serum #1 raised against immunogen-α and antiserum #4 against immunogen-β displayed a different reactivity (Fig. 2B). Antiserum #1 appeared to be devoid of context-unspecific antibodies and recognized a single 60-kDa protein band whether or not the serum was pre-incubated with inhibitor-T. Antiserum #4 recognized a band of the same size once the context-unspecific antibodies present in this serum had been neutralized. Interestingly, this 60-kDa band co-migrated with a band detected with pan anti-β-tubulin antibodies (Fig. 2B).

**Figure 3:**
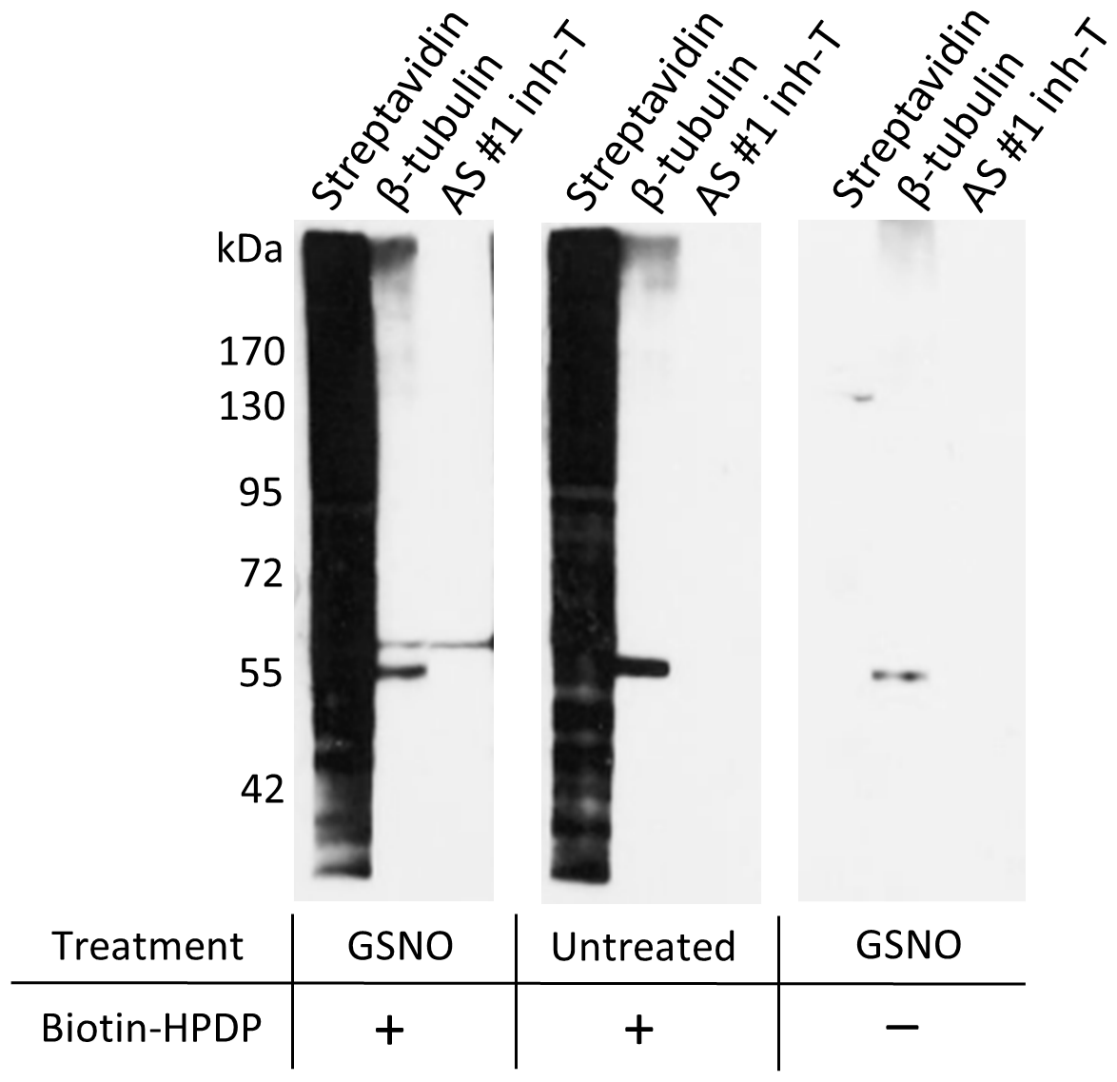
Detection of a 60-kDa band in GSNO treated protein lysates. Lysates of differentiated N2a cells were either treated with 100 µM GSNO or left untreated. After treatment the lysates were subjected to the biotin switch protocol in the presence or absence of biotin-HPDP as indicated. The resulting lysates were fractionated by non-reducing SDS-PAGE and analyzed by immunoblotting using antiserum #1 pre-incubated with inhibitor-T (*AS#1 inh-T*), streptavidin-HRP (*streptavidin*) and pan anti-β-tubulin antibodies (*β-tubulin*) as indicated.

These results raised the possibility that the 60-kDa band represented S-nitrosylated α- and/or β-tubulin and was specifically detected by antisera #1 and #4. We first tested whether detection of the 60-kDa band depended on S-nitrosylation. Lysates of differentiated N2a cells treated either with an NO donor (GSNO) or left untreated were subjected to the biotin switch protocol. We observed that detection of the 60-kDa band was contingent on treatment of the lysates with NO donor (Fig. 3). The lack of detection of the 60-kDa band in untreated sample correlated with the lack of detection of the co-migrating 60-kDa band detected by pan anti-β-tubulin antibodies, suggesting that the 60-kDa band indeed represented S-nitrosylated tubulin and not tubulin itself. As an additional control we used GSNO treated lysates that were subjected to the biotin switch protocol under omission of biotin-HPDP in the final step. Again the 60-kDa band was not observed with antiserum #1 and #4, suggesting that the 60-kDa band is a product of both GSNO treatment and subsequent application of the biotin switch protocol and not of GSNO treatment alone (Fig. 3). Surprisingly, pan anti-β-tubulin antibodies also failed to detect the 60-kDa band when labeling with biotin-HPDP was omitted (Fig. 3). This might indicate that subjecting S-nitrosylated tubulin to the biotin switch protocol causes an unexpected change in its electrophoretic mobility or that that the 60-kDa band does not represent S-nitrosylated β-tubulin and instead is an artifact of the method.

**Figure 4:**
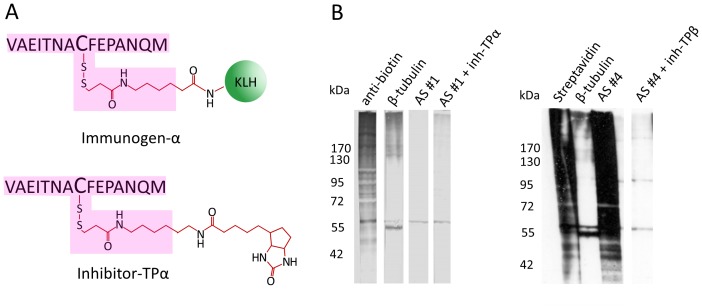
Detection of the 60-kDa band is insensitive to blocking of the antisera with specific inhibitor-TP. (A) Schematic representation of immunogen-α and the corresponding inhibitor-TPα. Pink shading outlines the epitope of interest. (B) Lysates of differentiated N2a cells were treated with 100 µM GSNO and then subjected to the biotin switch protocol. The resulting biotinylated lysates were fractionated by non-reducing SDS-PAGE and transferred to a nitrocellulose membrane. Following blocking the membrane was cut into stripes and each stripe was then probed with the indicated antiserum (*AS*) diluted at 1∶500 pre-incubated in the presence (*+ inh-TP*) or absence of inhibitor-TP. Successful application of the biotin switch protocol was confirmed with anti-biotin antibodies (*anti-biotin*) or streptavidin-HRP (*streptavidin*) and the position of tubulin bands was located with anti-β-tubulin antibodies (*β-tubulin*) as indicated.

To reveal the identity of the 60-kDa band and at the same time confirm the specificity of antisera #1 and #4, we generated biotin-HPDP modified peptides, termed inhibitor-TPα and inhibitor-TPβ, which mimic the modified cysteine epitopes on α- and β-tubulin, respectively, which were used as immunogens (Fig. 4A). These inhibitors should neutralize both context-specific and context-unspecific antibodies and block reactivity of the antisera towards all biotin-HPDP tagged proteins including α- and β-tubulin. However, neither of the inhibitors inhibited the detection of the 60-kDa band by the corresponding antiserum, not even at a molar excess of 500-fold over IgG concentration in the serum. These results suggested that the 60-kDa band, although detected only after treatment of protein lysates with GSNO followed by the biotin switch protocol and co-migrating with a band detected by pan anti-β-tubulin antibodies under the same conditions, most probably represents another protein and not S-nitrosylated α- and/or β-tubulin.

### Characterization of Antisera Raised Against Tagged Tubulin Peptides by Immunocytochemistry

**Figure 5:**
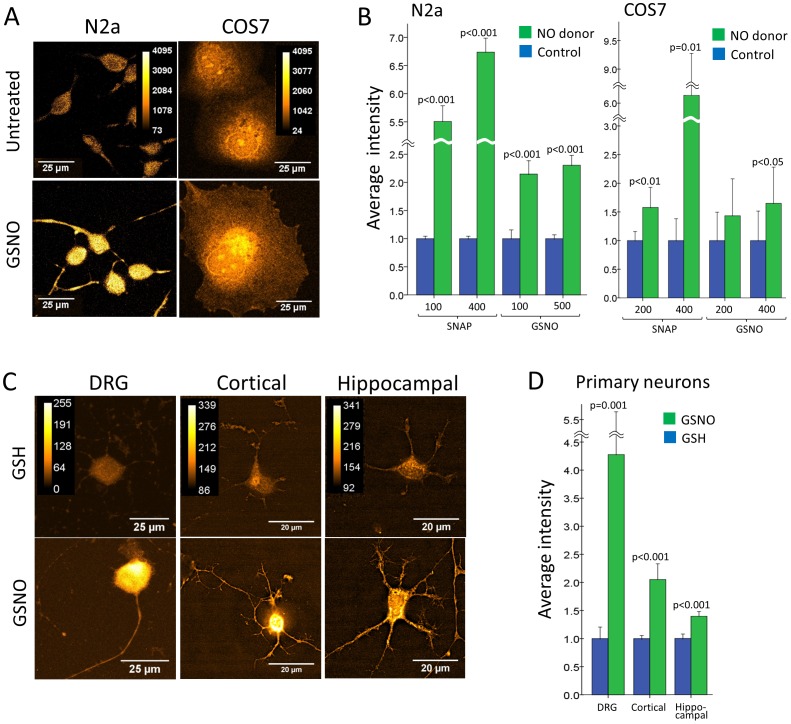
The *in situ* biotin switch assay reveals rapid increase in intracellular protein S-nitrosylation after treatment with NO donors. (A, B) *In situ* biotin switch assay on fixed cells of established cell lines. Differentiated N2a cells and COS7 cells were treated for 20 min with NO donor (SNAP or GSNO at the indicated µM concentrations), treated with solvent (control for SNAP treatment) or were left untreated (control for GSNO treatment). Following treatment, cells were fixed with cold methanol and subjected to the *in situ* biotin switch protocol. The resulting biotin-HPDP-tagged proteins were detected with streptavidin-rhodamine red. (A) Optical slices of stained cells. To visualize both high and low intensity areas, streptavidin-rhodamine red is shown using an orange-hot lookup table (LUT). For each cell type the corresponding high-low intensity color legend is shown. (B) Average intensity per cell normalized to average intensity of the control group (error bars 95% of confidence interval). Both cell types show a significant increase in the amount of S-nitrosylated proteins after treatment with NO donors (up to 6-fold). p values are indicated where the effect was significant. (C, D) *In situ* biotin switch assay in primary neurons. Primary cultures of adult DRG neurons (*DRG*), P0 cortical neurons (*Cortical*) or P0 hippocampal neurons (*Hippocampal*) were treated for 20 min with 500 µM GSNO or with 500 µM GSH as a control. Following treatment, cells were fixed with cold methanol, subjected to the *in situ* biotin switch protocol and stained with streptavidin-rhodamine red. (C) Optical slices of stained cells. To visualize both high and low intensity areas, streptavidin-rhodamine red is shown using an orange-hot LUT. For each cell type the corresponding high-low intensity color legend is shown. (D) Average intensity per cell normalized to average intensity of the control group (error bars 95% of confidence interval). All cell types show a significant increase in the amount of S-nitrosylated proteins after treatment with NO donor (up to 5-fold). p values are indicated.

The original aim of our study was to produce antibodies which specifically recognize S-nitrosylated tubulin *in situ* after fixation of the cells and replacement of the unstable nitroso moiety by a stable biotin-HPDP tag. Although the immunoblot analysis failed to demonstrate a specific detection of biotin-tagged tubulin (Fig. 4), it was of interest to test whether the antisera perhaps specifically detect tagged tubulin in fixed cells. For this purpose we first induced protein S-nitrosylation in cultured cells by treatment with NO donors. Both primary neurons and cells of established cell lines were treated with SNAP or GSNO to induce S-nitrosylation in living cells. Following fixation of the cells and application of a modified biotin switch protocol we detected a significant increase (up to 6 fold) in the amount of incorporated biotin compared to untreated cells (Fig. 5). These results suggested that bath application of NO donors led to rapid S-nitrosylation of intracellular proteins in all cell types used. Confocal microscopy did not reveal any filamentous structures and showed diffuse cytoplasmic staining (Fig. 5A, C).

**Figure 6:**
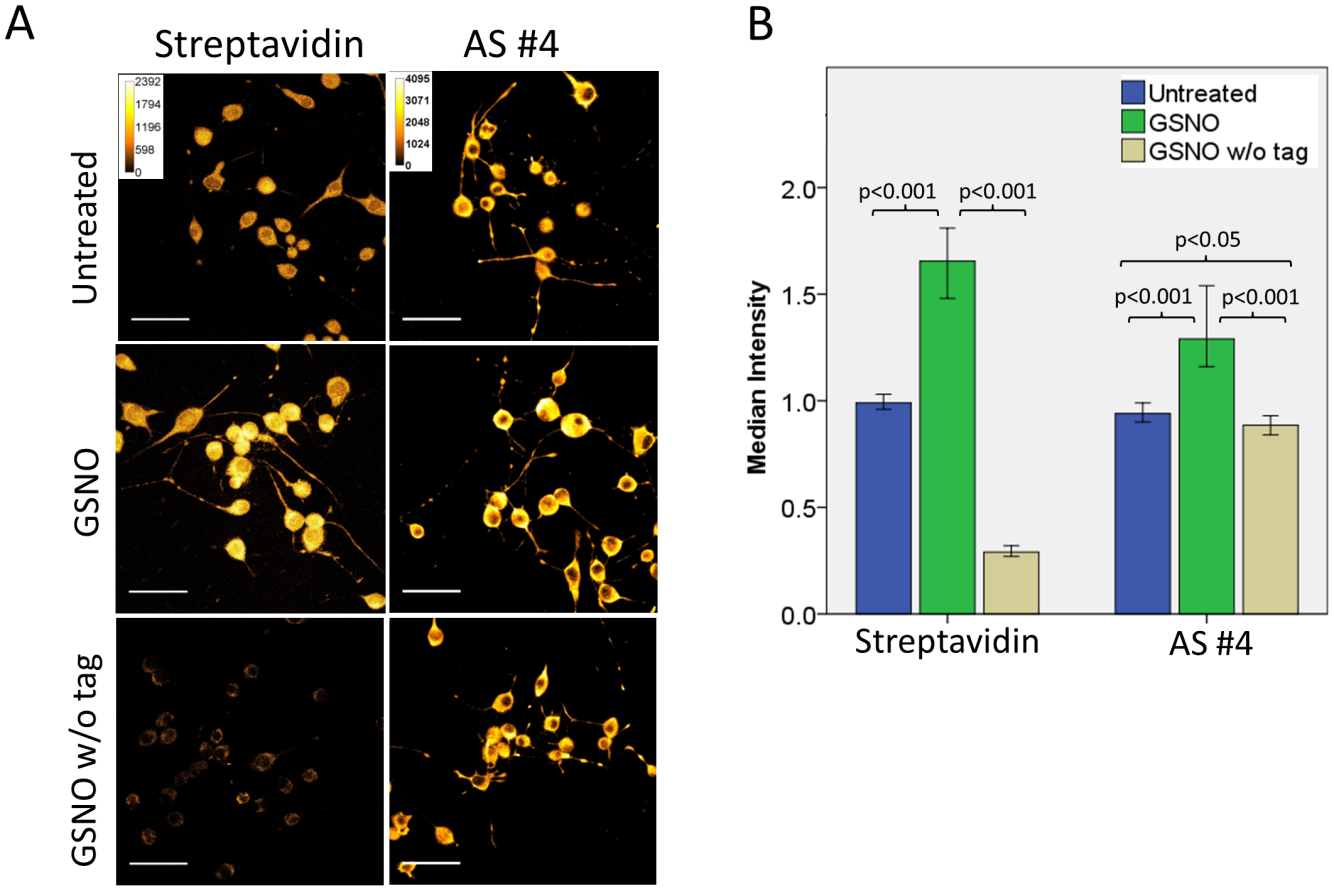
Immunological detection of protein S-nitrosylation in fixed cells. Differentiated N2a cells were treated for 20 min with 500 µM GSNO or left untreated as indicated. Following treatment, cells were fixed with cold methanol, subjected to the *in situ* biotin switch protocol and stained either with antiserum #4 (*AS #4*) or streptavidin-rhodamine red (*streptavidin*) as indicated. As a negative control, GSNO treated cells were subjected to the *in situ* biotin switch protocol without the biotin-HPDP tag (*GSNO w/o tag*). (A) Optical slices of stained cells. To visualize both high and low intensity areas, staining is shown using an orange-hot LUT. For each detection type (either streptavidin-rhodamine red or antiserum #4) the corresponding high-low intensity color legend is shown. (B) Median intensity per cell normalized to average intensity of the untreated group (error bars 95% of confidence interval). Cells treated with GSNO show a significant elevation of the signal when stained with antiserum #4, indicating the ability of the serum to detect labeled proteins. However, there is high background staining with antiserum #4 in negative control cells that were not labeled with tag. Scale bars, 50 µm.

The most promising antisera, antiserum #1 and #4, were tested for their capacity to detect biotin-HPDP tagged proteins in fixed cells. Differentiated N2a cells which showed the most robust response were treated with NO donor, subjected to the *in situ* biotin switch protocol and stained with antiserum #1 (data not shown) or antiserum #4 (Fig. 6). Quantification of the fluorescence intensity showed that pretreatment with NO donor caused a significant 1.4-fold increase (Fig. 6B). Quantification of the streptavidin-rhodamine red fluorescence intensity showed a similar increase (∼1.7 fold, Fig. 6B) indicating that context-unspecific antibodies could be responsible for a substantial part of the fluorescence increase after staining with antiserum #4. This is consistent with the observation that inhibitor-T blocked most of the reactivity of the antiserum against biotin-tagged proteins in immunoblots. Omission of the biotin-tagging step of GSNO treated cells revealed high background staining by the antiserum (Fig. 6). Finally, when context-unspecific antibodies were blocked by pre-incubation with the inhibitor-T, the staining with both antisera #1 and #4 was similar regardless of whether or not cellular proteins were modified with biotin-HPDP (data not shown). Taken together, these results suggested that the antisera contained context-unspecific antibodies capable of staining biotin-HPDP tagged proteins in fixed cells, but also produced a considerable level of background staining. Under no circumstances did we observe staining patterns indicative of microtubule staining.

## Discussion

The most recently discovered post-translational modification of tubulin is S-nitrosylation of α- and β-tubulin found in the murine brain [5]. Three cysteine residues in α-tubulin and 4 in β-tubulin have been identified to be susceptible to this modification [6],[7], and it has been reported that S-nitrosylation of tubulin alters its polymerization [11]. Because in the past the subcellular localization of post-translational tubulin or microtubule modifications has yielded important clues about their potential roles in neuronal cell biology we aimed to develop tools to localize S-nitrosylated tubulin isoforms in fixed cells. Our approach was to replace the labile nitroso moiety by a stable biotin tag in fixed cells *in situ* employing available protocols [17],[18] and subsequently use antibodies which recognize the tag specifically in the context of the tubulin polypeptide sequence flanking the cysteine residue of interest. Previous attempts to detect S-nitrosylated proteins in fixed cells were indiscriminative in that the detection method recognized all S-nitrosylated proteins and did not single out a specific protein or group of proteins [17] [18] [26]. Our approach was intended to localize a specific S-nitrosylated protein in fixed cells above a background of other S-nitrosylated proteins which are localized uniformly across the cell.

All 4 of the antisera we obtained contained antibodies that detected the biotin-HPDP tag on S-nitrosylated and subsequently tagged proteins in immunoblots irrespective of the peptide sequence context of the tagged cysteine residue. These antibodies could be neutralized by pre-incubation of the sera with the generic inhibitor-T. In addition, 2 of the 4 the antisera reacted with a 60-kDa band co-migrating with β-tubulin in N2a cell lysates that had been subjected to treatment with an NO donor and subjected to the biotin switch protocol replacing NO with the stable biotin-HPDP tag. Reactivity of the antisera with the 60-kDa band was retained when context unspecific anti-tag antibodies present in the sera were blocked with the inhibitor-T, a peptide unrelated to tubulin containing a biotin-HPDP tagged cysteine. Omitting the NO treatment or the biotin-HPDP labeling eliminated detection of the 60-kDa band. These observations were promising and suggested that the two sera might contain the intended antibodies specifically reacting with biotin-HPDP tagged tubulin isoforms. If the antisera were indeed specific and the 60-kDa band represented tagged tubulin isoforms, the reactivity of the sera should be blocked by inhibitor-TP, the tagged tubulin peptide representing the tubulin S-nitrosylation site which was used as immunogen. Unfortunately, this was not the case, not even when inhibitor-TP was used at 500-fold molar excess. We confirmed by mass spectrometry that inhibitor-TP was indeed the tubulin peptide modified with biotin-HPDP at the internal cysteine (not shown), ruling out that the failure to block reactivity of the antisera towards the 60-kDa band was due to flaws in the inhibitor-TP. Taken together these results demonstrated that the antisera contain antibodies that are specific for the biotin-HPDP tag and can be used for immunological detection of S-nitrosylated proteins that had been subjected to the biotin switch protocol. However, the antisera do not contain antibodies specific for biotin-HPDP tagged tubulin.

The 60-kDa band detected on the immunoblots with our antisera was also detected with a pan anti-β-tubulin antibody, but not with either anti-α-tubulin or anti-β3-tubulin antibodies (not shown). Detection of this band by pan anti-β-tubulin antibodies was contingent on NO treatment and application of the biotin switch protocol. These findings might suggest that the 60-kDa band is a β-tubulin isoform (not β3-tubulin) which incorporated the tag, because it was S-nitrosylated. However, as outlined above, this band is also detected in an unspecific manner by antisera #1 and #4. While detection of the 60-kDa band was dependent on NO treatment and tagging, it did not occur through the tag, because a) the band was detected by antisera #1 and #4 despite neutralization of anti-tag activity and b) pan anti-β-tubulin antibodies do not contain anti-tag antibodies at all. Remarkably, antisera #1 and #4 as well as pan anti-β-tubulin antibodies (but not anti-α-tubulin or anti-β3-tubulin antibodies) were polyclonal rabbit antibodies. Taken together, these results suggest that the 60-kDa band represents an abundant S-nitrosylated and hence biotin-HPDP tagged protein unrelated to tubulin which crossreacts with pan anti-β-tubulin antibodies and other polyclonal rabbit antisera in an unspecific manner.

In the course of this study we established the *in situ* biotin switch protocol which has previously been used only on a few non-neuronal cells and cell lines [17],[18],[26],[27] for the first time for neuroblastoma N2a cells and primary mouse neurons. Using streptavidin-rhodamine red as the detection reagent we found that protein S-nitrosylation can be induced in neurons in cell bodies, axons and dendrites with SNAP being more efficient than GSNO as an NO donor. Interestingly, the cell types vary in their response with N2a cells and DRG neurons displaying higher levels of S-nitrosylation compared to cortical and hippocampal neurons and COS7 cells. The staining we obtained in COS7 cells (nuclear and perinuclear ER-like as well as at the cell cortex) was similar to what has been reported for this cell type [27].

Using our antisera in immunocytochemical assays on cells treated with an NO donor, fixed and subjected to the *in situ* biotin switch protocol showed that the antisera can be used to specifically detect S-nitrosylated and hence biotin-HPDP tagged proteins, albeit indiscriminately. Compared to streptavidin-rhodamine red the antisera displayed a higher background staining. Nevertheless, these antisera might be of use for *in situ* or immunoblot detection of S-nitrosylated and subsequently tagged proteins in a biotin/streptavidin-independent manner. Because they were raised against an immunogen containing the spacer of biotin-HPDP but not biotin itself (green shaded epitope in Fig. 2A), they will recognize any protein modified by a tag containing this spacer.

Our approach intended to yield polyclonal antibodies for the specific recognition of S-nitrosylated and subsequently tagged tubulin was not successful. An alternative strategy would be to raise monoclonal antibodies instead. Although one might have to screen a large number of clones, because the possibility of obtaining context-specific monoclonal anti-tag antibodies is apparently very low, in case of success these antibodies would be specific and would not require purification. One problem with all these epitope-directed approaches that makes *in situ* detection of S-nitrosylated proteins much more difficult than, for example, phosphorylated or acetylated proteins, is the instability of S-nitrosylation. This necessitates the replacement of the nitroso moiety by a tag. The tagging reaction must be specific and efficient and must yield a stable tag that is resistant to subsequent processing for detection. Our study suggests that tagging with biotin-HPDP which is based on a disulfide bond fulfills all these requirements. We chose this strategy because of the availability of a matching pair of tagging molecule (biotin-HPDP) and the corresponding bifunctional crosslinker for generating the immunogen (LC-SPDP) sharing a common epitope attached to the cysteine under investigation (Fig. 1 and Fig. S1). Nevertheless, for future attempts to raise context-specific antibodies for the detection of S-nitrosylated proteins it might be advisable to employ other tagging and crosslinking strategies, for example, one based on a thioether bond instead of a disulfide linkage. Such a tag should be equally efficient and specific and would have the added advantages of being irreversible and stable even under reducing conditions.

Another strategy that one could explore to stain fixed cells that had been subjected to the *in situ* biotin switch protocol is to combine indiscriminate staining of biotin-HPDP tagged proteins by streptavidin conjugated to a donor fluorophore with specific staining of the protein of interest using available and specific antibodies which can be labeled with an acceptor fluorophore. Close proximity of the two fluorophores only at subcellular regions where the protein is S-nitrosylated could be observed by fluorescence resonance energy transfer (FRET) or fluorescence life time microscopy (FLIM). A potential caveat of this strategy lies in the inability to predict how donor and acceptor have to be positioned to permit the transfer of resonance energy. Thus, one would have to consider production of multiple variants of donors and/or acceptors to increase the chance of finding a successful pair.

## Supporting Information

Figure S1 **Immunogen synthesis and sequence conservation of tubulin isoforms at** ***in vivo*** **S-nitrosylation sites.** (A) The two-step reaction chosen to link synthetic tubulin peptides containing S-nitrosylation sites to a KLH carrier protein through LC-SPDP. First, free thiol groups of the carrier protein are blocked and LC-SPDP is covalently attached to amino groups of the carrier protein yielding the pyridyldithio-activated carrier *(Reaction A).* Subsequently, the free thiol residue of the single cysteine in the peptide of interest is linked to LC-SPDP *(Reaction B)* yielding the immunogen. The concomitant release of pyridine-2-thione from LC-SPDP can be monitored by measuring absorbance at a wavelength of 343 nm. (B) Alignment of different murine tubulin isoforms of α- (top) and β-tubulins (bottom). The sequences of the regions flanking the cysteines of interest which were found to be S-nitrosylated in the brain are shown (amino acids 278–318 and 276–316 for α- and β-tubulins, respectively). Sequences of the peptides chosen for immunogen synthesis are shown *(Peptide alpha and Peptide beta).* Green boxes indicate the corresponding sequences in the isoforms. Red shading indicates sequence divergence. Names of isoforms and the corresponding database entries are indicated.
(TIF)

Figure S2 **Treatment of protein lysates with the NO donor SNAP causes S-nitrosylation of α-tubulin.** Equal aliquots of brain protein lysates were treated for 1 h at room temperature in the dark either with SNAP (to S-nitrosylate target proteins in the lysate) or, for negative controls, with 20 mM ascorbate in the presence of 1% SDS or were exposed to daylight for 1 h (to reduce endogenous nitrosothiols) as indicated. After treatment the lysates were subjected to the biotin switch protocol combined with resin-assisted capture [23]. In this procedure, proteins that are S-nitrosylated are covalently linked to a thiol-reactive resin from which they can be eluted with dithiothreitol. One third of each eluate (*E*) and 1.5 µg aliquots (A) or 1 µg aliquots (B) of the input brain lysates (*I*) were fractionated by reducing SDS-PAGE and were analyzed by immunoblotting with anti-pan-α-tubulin antibodies. A low level of detection of α-tubulin in the eluate of the ascorbate or light treated lysates reveals the background of the method (negative control). In each case, treatment with SNAP caused a strong increase of α-tubulin found in the eluate.
(TIF)
