# Supplementary figures and images for: Difficulties in Generating Specific Antibodies for Immunohistochemical Detection of Nitrosylated Tubulins
Source: PLoS One. 2013 Jun 28;8(6):e68168. doi: 10.1371/journal.pone.0068168 (PMC3696116; doi:10.1371/journal.pone.0068168)

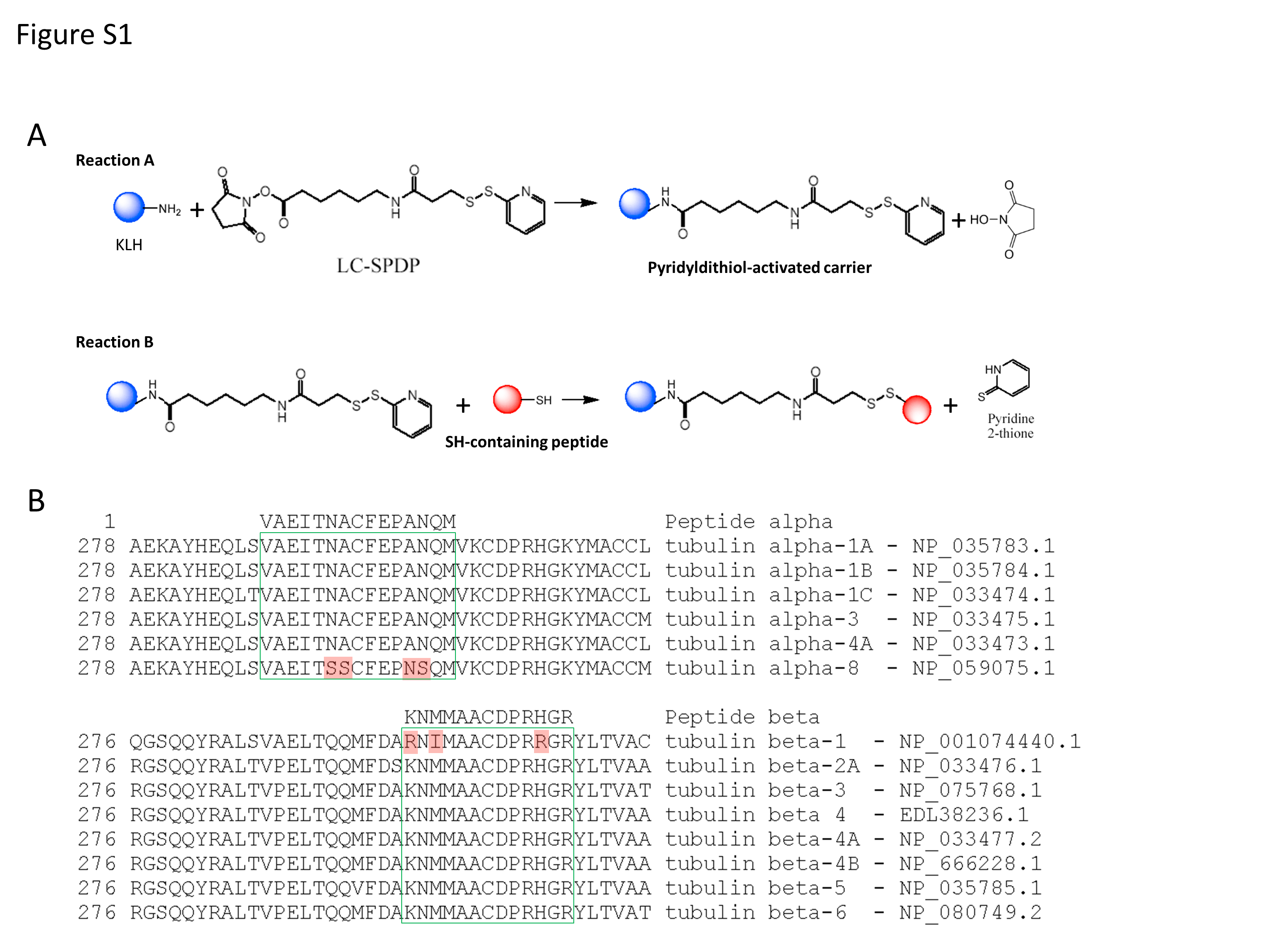

Supplement: Figure S1 — Immunogen synthesis and sequence conservation of tubulin isoforms at in vivo S-nitrosylation sites. (A) The two-step reaction chosen to link synthetic tubulin peptides containing S-nitrosylation sites to a KLH carrier protein through LC-SPDP. First, free thiol groups of the carrier protein are blocked and LC-SPDP is covalently attached to amino groups of the carrier protein yielding the pyridyldithio-activated carrier (Reaction A). Subsequently, the free thiol residue of the single cysteine in the peptide of interest is linked to LC-SPDP (Reaction B) yielding the immunogen. The concomitant release of pyridine-2-thione from LC-SPDP can be monitored by measuring absorbance at a wavelength of 343 nm. (B) Alignment of different murine tubulin isoforms of α- (top) and β-tubulins (bottom). The sequences of the regions flanking the cysteines of interest which were found to be S-nitrosylated in the brain are shown (amino acids 278–318 and 276–316 for α- and β-tubulins, respectively). Sequences of the peptides chosen for immunogen synthesis are shown (Peptide alpha and Peptide beta). Green boxes indicate the corresponding sequences in the isoforms. Red shading indicates sequence divergence. Names of isoforms and the corresponding database entries are indicated. (TIF) [file pone.0068168.s001.tif]
